# Correlation between the presumed pterygium with dry eye and with systemic and ocular risk factors

**DOI:** 10.5935/0004-2749.20220022

**Published:** 2025-08-21

**Authors:** Leidiane Adriano, Etiene Lorriane de Souza Persona, Isvander Gustavo de Souza Persona, Regina Celia Nucci Pontelli, Eduardo M. Rocha

**Affiliations:** 1 Departamento de Oftalmologia, Otorrinolaringologia e Cirurgia de Cabeça e Pescoço, Faculdade de Medicina de Ribeirão Preto, Universidade de São Paulo, Ribeirão Preto, SP, Brazil

**Keywords:** Pterygium/epidemiology, Dry-eye syndrome, Prevalence, Risk factors, Pterígio/epidemiologia, Síndrome do olho seco, Prevalência, Fatores de risco

## Abstract

**Objective:**

To estimate the epidemiology of the pterygium and its correlation with dry eye symptoms and with the potential systemic and ocular predictors.

**Methods:**

This study is a population-based, cross-sectional study in which random visits were made to the 600 households of 600 participants of age ≥40 years in Ribeirão Preto-SP (n=420) and Cassia dos Coqueiros-SP (n=180) in Brazil. The participants were subjected to a structured interview with a detailed questionnaire to collect information on demography and the potential risk factors. Next, random participants with pterygium (n=63) or not (n=110) were evaluated for the ocular surface changes.

**Results:**

The frequency of pterygium in Ribeirão Preto was 21% (15.7% among women and 32.1% among men; p=0.0002). In Cássia dos Coqueiros, the corresponding frequency was 19.4% (17.3% among women and 25.5% among men; p=0.28). The mean age of the affected individuals was higher than that of the unaffected ones (65.6 ± 10.5 years vs. 61.2 ± 12.0 years, p=0.02). A positive correlation was noted between pterygium and any prior radiotherapy and chemotherapy (p<0.0001, for both). A higher score on corneal fluorescein and conjunctival lissamine green staining was associated with pterygium (p=0.0003 and 0.0001, respectively).

**Conclusion:**

We noted a high frequency of pterygium in two Brazilian adult populations, mainly among the men and elderly. Ocular surface damage and a previous history of radiotherapy and/or chemotherapy were found to be associated with pterygium.

## Abstract (pt)

**Objetivo:**

Estimar a epidemiologia do pterígio; sua correlação com sintomas de olho seco e com potenciais preditores sistêmicos e oculares.

**Métodos:**

Estudo transversal, de base populacional, no qual foram realizadas visitas domiciliares aleatórias a 600 participantes, com 40 anos ou mais de idade, em Ribeirão Preto-SP (n=420) e Cassia dos Coqueiros-SP (n=180), Brasil. Uma entrevista estruturada com um questionário detalhado foi usada para coletar informações sobre demografia e possíveis fatores de risco. Em um segundo momento, participantes aleatórios com pterígio (n=63) ou não (n=110) foram avaliados quanto a alterações na superfície ocular.

**Resultados:**

A frequência de pterígio em Ribeirão Preto foi de 21%; 15.7% entre as mulheres e 32.1% entre os homens (p=0,0002). Em Cássia dos Coqueiros, essa taxa foi de 19.4%; onde 17.3% eram mulheres e 25.5% eram homens (p=0,28). A média de idade naqueles afetados pelo pterígio foi superior à dos participantes sem pterígio, 65,6 ± 10,5 e 61,2 ± 12,0 anos, respectivamente (p=0,02). Houve uma correlação positiva entre o pterígio e história prévia de radioterapia e quimioterapia (p<0,0001 para ambos). Houve maior coloração de fluoresceína na córnea e maior coloração de lissamina verde na conjuntiva em olhos com pterígio (p=0,0003 e 0,0001, respectivamente).

**Conclusão:**

Encontramos uma alta frequência de pterígio em duas populações adultas brasileiras, principalmente em homens e idosos. Danos na superfície ocular e história prévia de radioterapia e/ou quimioterapia foram associados ao pterígio.

## INTRODUCTION

Pterygium is a fibrovascular tissue of a triangular shape that grows from the peribulbar conjunctiva toward the cornea^(1)^.

A higher prevalence of pterygium has been reported in populations with high ultraviolet (UV) radiation exposure; this situation often related to working conditions, as demonstrated in the 3 studies conducted in China^(2-4)^, 1 in Australia^(5)^, and another 1 in India^(6)^.

A few Brazilian population studies have been published within reach of the world literature that may depict the prevalence of pterygium in the various regions of this continental country. A study conducted in Botucatu, Southeast Brazil that included individuals of both the sexes and across ages showed a prevalence of 8.12%, affecting most frequently 40-50-year-old men^(7)^. Two studies were conducted in the Brazilian Amazon: the first investigated 4 indigenous populations (Arawak, Tukano, Maku, and Yanomami), with adult participants of both the sexes, showing an 18.4% prevalence of pterygium, mainly in the Arawak and Tukano communities^(8)^; and the second study included subjects ≥45 years of age from the urban and rural areas of the Parintins city that showed a pterygium prevalence of 58.8%^(9)^.

Advanced age and male sex were frequently found to be associated with pterygium in previous studies conducted worldwide^(5,10-15)^. However, in other researches, no correlations had been made between pterygium and sex^(2,16,17)^. Similarly, some other studies showed no correlation between pterygium and age^(8,18)^. These contradictory results may be biased by other factors such as the lifestyle, climate, and exposure to environmental risk factors among distinct populations. Therefore, there remains controversies among pterygium frequency, demography, and its risk factors. Table 1 displays the summary of population studies on pterygium prevalence, the risk factors, and factors without any association.

**Table 1:** Summary of the cross-sectional studies published up to 20 years before the current paper: the country where the study was realized, the number of participants (N), age, pterygium prevalence, risk factors for pterygium, and factors without association to pterygium

Author(s), year	Country	N	Age (years)	Prevalence (%)	Associated factors	Non associated factors
McCarty CA et al, 2000^(5)^	Australia	5147	40-101	2.8	Older age, male sex, rural residence, smoking, cataract, take vitamin C, and outdoor work	Take vitamin E
Gazzard G et al, 2002^(19)^	Indonesia	1210	≥21	10	Older age, outdoor work, visual acuity, and astigmatism	Sex and smoking
Tan CSH et al, 2006^(13)^	Indonesia	477	All age	17	Older age and male sex	NI†
Paula JS et al, 2006^(8)^	Brazil	624	Adults	18.4	Arawak and Tukano communities	Sex and age
Lu J et al, 2009 ^(20)^	China	2486	≥40	17.9	Older age, cataract, low education level, alcohol, low	Sex
					income family situation, seldom use of sunglasses and	
					hat	
Shiratori CA et al, 2010^(7)^	Brazil	2554	All age	8.1	Male sex and age range 40-50 years	NI†
Landers J et al, 2011^(16)^	Australia	1884	≥20	7.8	Older age	Sex, visual acuity, cataract,
						and climatic keratopathy
Viso E et al, 2011^(21)^	Spain	1155	≥40	5.9	Older age and outdoor work	Sex, education level, iris
						color, smoking, alcohol,
						rosacea, allergy, and
						diabetes
Zhong H et al, 2012^(4)^	China	2133	≥50	39	Older age, female sex, low education level, visual acuity,	Diabetes, smoking,
					height, weight, hypertension, and outdoor work	and alcohol
Ang M et al, 2012^(10)^	Singapore	8906	40-80	10.1	Older age, male sex, Malay ethnicity, low education	Low-density or high-
					level, hypertension, hypercholesterolemia	density lipoprotein
						cholesterol levels, alcohol,
						diabetes, glycosylated
						hemoglobin A1c level, and
						previous ocular trauma
Rezvan F et al, 2012^(11)^	Iran	5190	≥40	9.4	Male sex, older age, outdoor work,	Smoking and visual acuity
					astigmatism, and low education level	
Tano T et al, 2013^(17)^	Japan	2312	40-74	4.4	Older age	Sex, outdoor work,
						and smoking
Marmamula S et al, 2013^(6)^	India	10293	≥30	11.7	Older age, rural residents, no education, outdoor work,	Sex and smoking
					and alcohol	
Nangia V et al, 2013^(12)^	India	4711	≥30	12.9	Older age, male sex, low education level, height,	DBP‡ and alcohol use
					weight, hypertension, smoking, BMI^*^, and outdoor work	
Jiao W et al, 2014^(3)^	China	17816	≥50	10.5	Older Age, low education level, outdoor time (hours per	Sex, alcohol, smoking,
					day), and wearing hat and/or sunglasses	diabetes, hypertension,
						hyperlipidemia, and
						cardiac diseases
Chen T et al, 2015^(2)^	China	4617	≥30	11.9	Older age, rural residence, astigmatism, low education	Sex, BMI^*^, diabetes,
					level, outdoor work, height, weight, hypertension, no	and alcohol
					exercise, heavy physical work, Han ethnicity, and smoking	
Mcknight CM et al, 2015^(22)^	Australia	1344	18-22	1.2	Male sex	Age, height, weight,
						education level
Pyo EY et al, 2016^(14)^	South	9193	≥40	8.8	Older age, male sex, rural residence, low education	Alcohol, BMI^*^, diabetes,
	Korea				level, low income, sunlight exposure, and hypertension	and hypercholesterolemia
Hashemi H et al, 2017^(23)^	Iran	3312	All age	13.1	Older age	Sex, education level
Fernandes, A. G, 2019^(9)^	Brazil	2041	≥45	58.8	Male sex, older age, and rural residence	Higher education was a
						protective factor

* = Body Mass Index; †= Not investigated; ‡= Diastolic blood pressure.

The present study aimed to estimate the pterygium frequency in 2 cities of the São Paulo State (in the tropical area of Brazil) and the correlation between this health issue and the ocular surface findings, as well as the ocular and systemic risk factors.

## METHODS

### Type and location of the study

We conducted a cross-sectional field study in 2 municipalities of Brazil through visits to the homes of randomly selected participants.

The municipality of Ribeirão Preto is located northwest of the capital of the state of São Paulo at the latitude 21^o^10’39”S; longitude 47^o^48’37”W; altitude 546 m; and regional area 650.916 km^2^. According to the latest demographic census (2010), the population aged ≥40 years residing in Ribeirão Preto was 226.462, of which 101.721 were men and 124.741 were women^(23)^.

The municipality of Cássia dos Coqueiros located in the southeastern region of Brazil, in the metropolitan area of Ribeirão Preto, has the latitude of 21^o^16’58’’S, longitude of 47^o^10’11’’ W, altitude of 890 m, and territorial area of 191.683 km^2^. According to the latest demographic census (2010), the population of age ≥40 years residing in Cássia dos Coqueiros was 1099, of which 567 are men and 532 are women^(24)^.

### Sample and inclusion criteria

The present study is a part of the original research on dry eye epidemiology in Brazil, in which we visited 600 participants at their residences, including 420 inhabitants belonging to Ribeirão Preto-São Paulo and 180 to Cássia dos Coqueiros-São Paulo of age ≥40 years and of both the sexes.

The sample was calculated using the formula for simple random sampling, as follows: n=Z2 [P (1 - P)] / D2.

Where, n is the sample size, P is the expected prevalence (assumed to be 10%), and D is the maximum acceptable error, adopted by 1.5% in the estimate. Of the total 400, 30% of the sample size found in the calculation was added, providing for refusals and/or withdrawals from the selected participants.

The exclusion criteria were a refusal to agree with the study conditions or provision of the informed consent. All types of pterygium (i.e., primaries, secondaries, or a history of surgical pterygium excision) were included for the calculi of the frequency.

### Material and diagnostic criteria

First, all participants were assessed for the analysis of lesions suggestive of pterygium. The ocular injury was characterized as pterygium if fibrovascular growth extending from the conjunctiva toward the limbus and enveloping the cornea was noted^(16)^. The collection of data from the research was performed under the supervision of an ophthalmologist.

A detailed questionnaire interview was applied to collect information about the presence of 12 systemic factors, namely, 1. sex, 2. age, 3. diabetes mellitus, 4. woman post-menopause, 5. rheumatic diseases, 6. a previous history of leprosy, 7. treatment with chemotherapy, 8. treatment with radiotherapy, 9. thyroid diseases, 10. the use of antidepressants, and/or 11. the use of antiallergic chronically or within the last 30 days, and 12. dyslipidemia; as well as 4 ocular factors, namely, 1. a history of trachoma, 2. past ocular surgeries (such as cataract and blepharoplasty), 3. the use of contact lens, and 4. the use of the computer or cell phone for at least 2 hours in a day; in addition to the symptoms of dry eye disease (DED) (Appendix 1).

**Appendix 1:** Questionnaires applied in the dry eye epidemiology research in Brazil

Portuguese version	English version
1. Você sente os olhos secos?	1. Do you feel your eyes dry?
() Nunca () Raramente () Frequentemente () Sempre	() Never () Rarely () Often () Always
2. Você sente os seus olhos irritados?	2. Do you feel your eyes irritated?
() Nunca () Raramente () Frequentemente () Sempre	() Never () Rarely () Often () Always
3. Você já teve diagnóstico de olho seco?	3. Have you ever had a dry eye diagnosis?
() Sim () Não	() Yes () No
**a) Dry eye symptom questionnaire: Portuguese and English versions**	
**Systemic**	**Ocular**
Diabetes mellitus	Eye surgery
Postmenopausal women	Computer and/or mobile 2 hours or more daily
Rheumatological diseases	Contact lens
Leprosy	Trachoma
Chemotherapy	Pterygium
Radiotherapy	
Thyroid disease	
Antidepressants	
Antiallergic	
Chronic Pelvic Pain	
Fibromyalgia	
Dyslipidemia	

In the second phase of the study, 63 participants with lesions suggestive of pterygium to ectoscopy were evaluated at a health center and compared to 110 healthy controls without any previous conjunctival eye surgery. For this purpose, all participants who presented with symptoms of DED and 1 in every 5 participants with no symptoms were invited; based on the responses from the brief, previously described questionnaire^(25,26)^.

The Schirmer test 1 (ST) was performed using a strip of filter paper (Ophthalmos Ltd., SP, Brazil) and the wetting of the tape was observed after 5 min of placing it on the temporal third of the patient’s lower eyelid without anesthesia.

The tear film breakup time (TFBUT) and corneal fluorescein staining (CFS) were determined by instilling a drop of 1% sodium fluorescein eye drops (Ophthalmos Ltd.) to check the tear break between a blink and the other eyelid and then to quantify the CFS following the 15-point National Eye Institute (NEI)/Industry scale (grades 0-3 for 5 regions of the ocular surface).

The lissamine green conjunctival staining was evaluated after contacting the participant’s lower eyelid sac with a strip of paper dipped in lissamine green (Ophthalmos Ltd.). Following the classification described by van Bijsterveld (modified NEI/industry scale), 3 conjunctival regions (i.e., temporal, central, and nasal) received scores from 0 to 3.

The Ocular Surface Disease Index (OSDI) was quantified using an OSDI Portuguese validated version^(27)^.

The following findings were considered with the signs of DED: ST 1 £10 mm, TFBUT <10 s, CFS >3, and CLGS >3. The presence of dry eye symptoms was considered on identifying a positive result on the Short Questionnaire or an OSDI score >13^(26,28)^.

The data collection from the questionnaires and clinical examinations were conducted in July 2016 in Cássia dos Coqueiros and from the end of June to August 2017 in Ribeirão Preto. As such, the whole research was conducted in the winter season in the state of São Paulo, Brazil.

### Ethical considerations

The research followed the tenets of the Declaration of Helsinki, and the local Research Ethics Committee approved the research project. All participants were informed about the content and purpose of the research and their signed Free and Informed Consent Term form was obtained.

### Statistical analysis

The Access Database 2016 (Microsoft Corporation, Seattle, Washington, USA) was used to record and store the survey data.

The GraphPad Prism 5.1 software was used for statistical calculations. For statistical analyses, we used the Fischer’s exact test, *t*-test, Mann-Whitney U-test, and percentage. The acceptable p value was <0.05 (95% CI).

## RESULTS

The frequency of pterygium in the adult population of Ribeirão Preto was 21% (out of 420 participants). The frequency of pterygium in Cássia dos Coqueiros was 19.4% (out of 180 participants).

The frequency of pterygium in women in Ribeirão Preto was 15.7% (out of 286 participants), while it was 32.1% (out of 134 participants) in men (p=0.0002).

In the municipality of Cássia dos Coqueiros, the frequency of pterygium among women was 17.3% (out of 33 participants), while it was 25.5% (out of 47 participants) among men (p=0.28).

Including the 600 participants from the 2 cities involved in the study, the mean age in those affected by pterygium was 65.6 ± 10.5 years, which indicated a significant difference from those without pterygium (61.2 ± 12.0 years); p=0.02, unpaired t-test.

The frequency of pterygium location (i.e., nasal or temporal) for the 2 cities evaluated together was 121 cases nasal (98.4% of the 123) and 2 temporal cases (1.6% of the 123). The graduation of pterygium according to its size was not quantified. Thirty-four cases (27.6% of the 123) were found to be bilateral, and all of them were nasal.

We investigated the correlation among the pterygium, systemic, and ocular factors, and the following factors were not associated with pterygium: diabetes mellitus (n=123, p=0.42, OR=0.81, 95%CI=0.51-1.29); post-menopause women (n=53, p=0.30, OR=1.45, 95%CI= 0.78-2.68); rheumatic diseases (n=17, p=1.00, OR= 0.95, 95%CI=0.53-1.68); leprosy (prior or active) (n=2, p=0.19, OR=3.93, 95%CI= 0.55-28.17); thyroid diseases (n=20, p=0.47, OR= 1.21, 95%CI=0.70-2.08); the use of antidepressants chronically or within the last 30 days (n=29, p=1.00, OR=0.99, 95%CI= 0.62-1.58); the use of antiallergic agents chronically or within the last 30 days; dyslipidemia (n=38, p=0.83, OR= 0.95, 95%CI= 0.62-1.45); a history of trachoma (n=1, p=1.00, OR= 0.64, 95%CI = 0.08-5.40); past ocular surgeries (such as cataract, blepharoplasty, and vitrectomy) (n=33, p=0.56, OR=1.17, 95%CI =0.74-1.83); the use of contact lens (n=1, p=1.00, OR=0.64, 95%CI =0.08-5.40); and the use of computer or cell phone for at least 2 hours in a day (n=17, p=0.54, OR=1.18, 95%CI = 0.66-2.11). The following factors were positively correlated: chemotherapy (n=2, p<0.0001, OR=30.08, 95%CI=8.91-101.6) and any form of radiotherapy (n=2, p<0.0001, OR=30.08, 95%CI=8.91-101.6) (Table 2).

**Table 2:** Evaluation of the association among pterygium and systemic and ocular factors in 2 Brazilian cities, participants number (N) = 600

Factor (N)	N with PT^*^ (%)	OR ^†^ (95% CI)	Fisher’s exact test p value
Systemic			
Female (419)	68 (16.23)	0.44 (0.29-0.67)	0.0002
Male (181)	55 (30.39)	2.25 (1.50-3.39)	0.0002
Dibetes (160)	123 (76.88)	0.81 (0.51-1.29)	0.42
Women menopause (302)	53 (17.55)	1.45 (0.78-2.68)	0.30
Rheumatic disease (86)	17 (19.77)	0.95 (0.53-1.68)	1.00
Past of leprosy (4)	2 (50)	3.93 (0.55-28.17)	0.19
Chemotherapy (24)	2 (8.33)	30.08 (8.91-101.6)	<0.0001
Radiotherapy (24)	2 (8.33)	30.08 (8.91-101.6)	<0.0001
Thyroid disease (86)	20 (23.25)	1.21 (0.70-2.08)	0.47
Oral antidepressants (142)	29 (20.42)	0.99 (0.62-1.58)	1.00
Oral Antiallergics (60)	11 (18.33)	0.86 (0.43-1.70)	0.74
Dyslipidemia (191)	38 (19.89)	0.95 (0.62-1.45)	0.83
Ocular			
Contact lens use (7)	1 (14.28)	0.64 (0.08-5.40)	1.00
Ocular surgery (80)	33 (41.25)	1.17 (0.74-1.83)	0.56
Past of trachoma (7)	1 (14.28)	0.64 (0.08-5.40)	1.00
Computer or cell phone 2h or more/day (74)	17 (22.97)	1.18 (0.66-2.11)	0.54

* = Pterygium; †= Odss ration.

**Figure 1:**
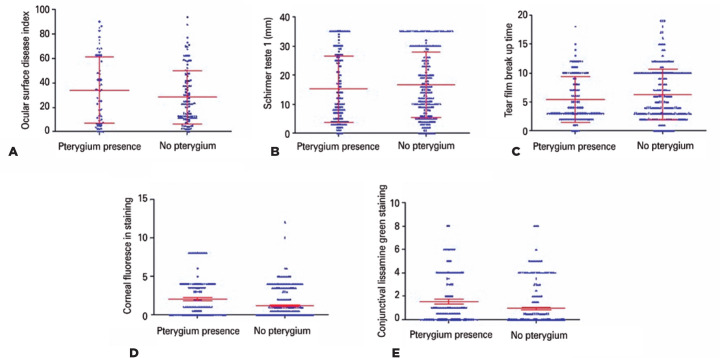
Comparison of the ocular surface tests between pterygium (n=63) and control individuals (n=110).

The following ocular surface tests were not different between the individuals with pterygium and the control subjects (OSDI, p=0.35; ST without anesthesia, p=0.11; TFBUT, p=0.10). A difference was noted among individuals with pterygium and the control subjects in the following ocular surface tests (CFS, p=0.0003; and CGLS, p=0.0001) (Figure 1).

**Figure 2:**
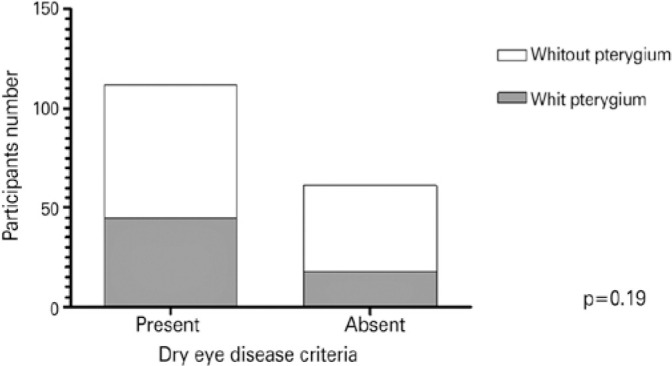
Comparison between dry eye disease frequency between the pterygium group and control group.

Of the 173 participants evaluated in the second phase of the research, 112 (64.7%) presented with criteria for DED, as described in the methodology. No difference was noted in the frequency of DED between the pterygium and the control groups (p=0.19, OR=1.60, 95%CI=0.82-3.13), as demonstrated in figure 2.

## DISCUSSION

Evaluation of the prevalence of pterygium in the 4 studies involving Brazilian populations, including the present study, showed a variation of 8.1%-58.8%^(7-9)^. One of the explanations for the lower frequency of pterygium recorded in the Botucatu City is that the study covered individuals of all ages, and pterygium is known to occur more commonly at older age, which widens the variation of pterygium frequency in the studies conducted among the Brazilian populations.

This study demonstrated a higher frequency of pterygium among men, which agrees with a large number of studies conducted across the world^(5,10-12)^, including researches performed in Brazil^(7,9)^. However, in contracts, a Chinese study noted a correlation between pterygium and female sex among participants aged ≥50 years^(4)^. No statistically significant association was noted with pterygium and sex in a study conducted in India in 2013^(6)^ as well as in 3 studies conducted in China^(2,3,20)^, 1 in Japan ^(17)^, 1 in Indonesia^(19)^, 1 in Spain^(21)^, and 1 in Brazil^(8)^. In the present study, interestingly, the protective effect of the female sex was lost by menopause when the frequency of pterygium reached nearer to that of men.

The average age of the participants with pterygium was significantly higher than the mean age of the participants without this ocular lesion, which agrees with the results of several other studies from across the world^(2-5,16,21,23)^. However, no relationship was noted between older age and pterygium in several studies, such as that conducted in Australia in 2015^(22)^ and that conducted in Brazil in 2006^(8)^.

In the total sample, the most frequent location of typical pterygium lesion was nasal, as was expected, which agrees with the reposts of other epidemiological studies^(2,22)^.

No statistical correlation was noted between pterygium and diabetes, which agrees with the results of the 3 studies conducted in China^(2-4)^, 1 in Singapore^(10)^, 1 in South Korea^(14)^, and 1 in Spain^(21)^.

In addition, no correlation was noted between pterygium and dyslipidemia. A similar result was noted in other studies^(3,14)^. In contrast, a study conducted in Singapore identified an association between pterygium and hypercholesterolemia^(10)^.

However, no correlation was recorded between pterygium and the use of antiallergic agents, which agrees with the result of a Spanish study in which no correlation was established between allergy and pterygium^(14)^.

Moreover, no statistical correlation was established among pterygium and rheumatic diseases, a history of leprosy, thyroid diseases, the use of antidepressants, a history of trachoma, past ocular surgeries (such as cataract and blepharoplasty), the use of contact lens, and the use of a computer or cell phone for at least 2 hours in a day, which are the factors that are otherwise not frequently investigated in the literature.

A recent population study conducted in China recorded that premature menopause is associated with the presence of pterygium of grade ≥2, which is different from the findings of the present study, in which no statistical correlation was recorded between pterygium and postmenopausal woman. Therefore, more studies are needed to justify the association reported in the present study, as there are no previous studies that investigated this relationship^(29)^.

Among the 24 patients with a history of anti-neoplastic treatment, a positive correlation was noted among pterygium, chemotherapy, and radiotherapy (n=2, 8.3% of the 24 participants). The authors hence sought to investigate the association between pterygium and a history of treatment with chemotherapy and/or radiotherapy because this assessment had remained unexplored in previous studies. Moreover, these interventions have been associated with side effects that include dry eye. Although dry eye is an entirely common symptom among the adult population, the present study intended to identify more specific correlations. The possible mechanism of this association between chemotherapy or radiotherapy with pterygium must be dry eye and ocular surface chronic inflammation, albeit it requires further confirmation.

During the evaluation of the correlation between pterygium and DED criteria, no difference was noted between the pterygium group and the control group. However, an association was recorded with the staining tests CFLS and CGLS. Similarly, a study conducted in Spain in 2011 also noted no association with dry eye symptoms or similar signs, except for that by fluorescein staining^(21)^. In contrast, a Chinese study in 2009 identified an association between pterygium and lower Schirmer’s test, tear breakup time, and the presence of dry eye symptoms^(20)^.

The present study has some limitations: it includes differential diagnoses of lesions that mimic pterygium and that cannot be identified by ectoscopy or by examination under a slit lamp in addition to the issue of the lack of demographic information regarding UV exposure, work activity, and the educational levels of participants that allows comparison with previous studies and the identification of some possible confounding factors that have not been explored in this study.

The present study employed a questionnaire designed to investigate factors associated with DED, which did not include all the variables assessed in previous studies for pterygiu. Our results can hence mislead the association between exposure and outcome (cause and effect) and indicate as causes items that are only associated with the real causes. Future studies are therefore necessary to investigate these possibilities in further detail.

In conclusion, the frequency of pterygium in the 2 Brazilian populations was similar and high, approximately 20%, more frequently among men and older people. Fluorescein staining and white lissamine green staining were associated with pterygium. A positive correlation between pterygium and a history of chemotherapeutic and radiotherapeutic treatment was noted. However, there was a large discrepancy in the risk factors associated with pterygium, both in the epidemiological context (such as with respect to the sex and age) and clinical factors (such as diabetes and dyslipidemia) on comparing our findings with other studies, which emphasizes on the need for more comprehensive studies on this subject.

## References

[1] Jaros PA, DeLuise VP. (1988). Pingueculae and pterygia. Surv Ophthalmol.

[2] Chen T, Ding L, Shan G, Ke L, Ma J, Zhong Y. (2015). Prevalence and racial differences in pterygium: a cross-sectional
study in Han and Uygur adults in Xinjiang, China. Invest Ophthalmol Vis Sci.

[3] Jiao W, Zhou C, Wang T, Yang S, Bi H, Liu L (2014). Prevalence and risk factors for pterygium in rural older adults
in Shandong Province of China: a cross-sectional study. BioMed Res Int.

[4] Zhong H, Cha X, Wei T, Lin X, Li X, Li J (2012). Prevalence of and risk factors for pterygium in rural adult
Chinese populations of the Bai nationality in Dali: the Yunnan Minority Eye
Study. Invest Ophthalmol Vis Sci.

[5] McCarty CA, Fu CL, Taylor HR. (2000). Epidemiology of pterygium in Victoria, Australia. Br J Ophthalmol.

[6] Marmamula S, Khanna RC, Rao GN. (2013). Population-based assessment of prevalence and risk factors for
pterygium in the South Indian state of Andhra Pradesh: the Andhra Pradesh
Eye Disease Study. Invest Ophthalmol Vis Sci.

[7] Shiratori CA, Barros JC, Lourenço RM, Padovani CR, Cordeiro R, Schellini SA. (2010). [Prevalence of pterygium in Botucatu city - São Paulo
State, Brazil]. Arq Bras Oftalmol.

[8] Paula JS, Thorn F, Cruz AA. (2006). Prevalence of pterygium and cataract in indigenous populations of
the Brazilian Amazon rain forest. Eye (Lond).

[9] Fernandes AG, Salomao SR, Ferraz NN, Mitsuhiro MH, Furtado JM, Munoz S (2020). Pterygium in adults from the Brazilian Amazon Region: prevalence,
visual status and refractive errors. Br J Ophthalmol.

[10] Ang M, Li X, Wong W, Zheng Y, Chua D, Rahman A (2012). Prevalence of and racial differences in pterygium: a multiethnic
population study in Asians. Ophthalmology.

[11] Rezvan F, Hashemi H, Emamian MH, Kheirkhah A, Shariati M, Khabazkhoob M (2012). The prevalence and determinants of pterygium and pinguecula in an
urban population in Shahroud, Iran. Acta Med Iran.

[12] Nangia V, Jonas JB, Nair D, Saini N, Nangia P, Panda-Jonas S. (2013). Prevalence and associated factors for pterygium in rural agrarian
central India. The central India eye and medical study. PLoS One.

[13] Tan CS, Lim TH, Koh WP, Liew GC, Hoh ST, Tan CC (2006). Epidemiology of pterygium on a tropical island in the Riau
Archipelago. Eye (Lond).

[14] Pyo EY, Mun GH, Yoon KC. (2016). The prevalence and risk factors for pterygium in South Korea: the
Korea National Health and Nutrition Examination Survey (KNHANES)
2009-2010. Epidemiol Health.

[15] Lim CY, Kim SH, Chuck RS, Lee JK, Park CY. (2015). Risk factors for pterygium in Korea: The Korean National Health
and Nutrition Examination Survey V, 2010-2012. Medicine (Baltimore).

[16] Landers J, Henderson T, Craig J. (2011). Prevalence of pterygium in indigenous Australians within central
Australia: the Central Australian Ocular Health Study. Clin Exp Ophthalmol.

[17] Tano T, Ono K, Hiratsuka Y, Otani K, Sekiguchi M, Konno S (2013). Prevalence of pterygium in a population in Northern Japan: the
Locomotive Syndrome and Health Outcome in Aizu Cohort Study. Acta Ophthalmol.

[18] Gazzard G, Saw SM, Farook M, Koh D, Widjaja D, Chia SE (2002). Pterygium in Indonesia: prevalence, severity and risk
factors. Br J Ophthalmol.

[19] Lu J, Wang Z, Lu P, Chen X, Zhang W, Shi K (2009). Pterygium in an aged Mongolian population: a population-based
study in China. Eye (Lond).

[20] Viso E, Gude F, Rodríguez-Ares MT. (2011). Prevalence of pinguecula and pterygium in a general population in
Spain. Eye (Lond).

[21] McKnight CM, Sherwin JC, Yazar S, Forward H, Tan AX, Hewitt AW (2015). Pterygium and conjunctival ultraviolet autofluorescence in young
Australian adults: the Raine study. Clin Exp Ophthalmol.

[22] Hashemi H, Khabazkhoob M, Yekta A, Jafarzadehpour E, Ostadimoghaddam H, Kangari H. (2016). The prevalence and determinants of pterygium in rural
areas. J Curr Ophthalmol.

[23] Instituto Brasileiro de Geografia e Estatística
(IBGE) (2020). Censo demográfico 2010: Ribeirão Preto, 2010.

[24] Instituto Brasileiro de Geografia e Estatística
(IBGE) (2020). Censo demográfico 2010: Cássia dos Coqueiros,2010.

[25] Gulati A, Sullivan R, Buring JE, Sullivan DA, Dana R, Schaumberg DA. (2006). Validation and repeatability of a short questionnaire for dry eye
syndrome. Am J Ophthalmol.

[26] Castro JS, Selegatto IB, Castro RS, Vasconcelos JP, Arieta CE, Alves M. (2017). Translation and validation of the Portuguese version of a dry eye
disease symptom questionnaire. Arq Bras Oftalmol.

[27] Prigol AM, Tenório MB, Matschinske R, Gehlen ML, Skare T. (2012). [Translation and validation of ocular surface disease index to
Portuguese]. Arq Bras Oftalmol.

[28] Castro JS, Selegatto IB, Castro RS, Miranda EC, de Vasconcelos JP, de Carvalho KM (2018). Prevalence and Risk Factors of self-reported dry eye in Brazil
using a short symptom questionnaire. Sci Rep.

[29] Pan Z, Cui J, Shan G, Chou Y, Pan L, Sun Z (2019). Prevalence and risk factors for pterygium: a cross-sectional
study in Han and Manchu ethnic populations in Hebei, China. BMJ Open.

